# Glucose and Lipid Metabolic Mechanisms in Vascular Aging and Related Therapeutic Strategies

**DOI:** 10.31083/RCM45053

**Published:** 2026-04-08

**Authors:** Fei Han, Zuoshi Wen, Chenxi Li, Ting Chen

**Affiliations:** ^1^Department of Cardiology, The First Affiliated Hospital, College of Medicine, Zhejiang University, 310003 Hangzhou, Zhejiang, China; ^2^Key Laboratory of Precision Medicine for Atherosclerotic Diseases of Zhejiang Province, The First Affiliated Hospital of Ningbo University, 315000 Ningbo, Zhejiang, China

**Keywords:** vascular aging, glucose, lipid metabolism, multi-omics

## Abstract

Vascular aging represents an independent risk factor for vascular disorders, with underlying cellular and molecular mechanisms closely associated with metabolic dysfunction, particularly disturbances in glucose and lipid metabolism. Therefore, understanding the interplay between disorders in glucose and lipid metabolism, as well as vascular aging, is crucial for preventing vascular disease. Early clinical recognition and targeted intervention are essential for the diagnosis and management of vascular senescence-related conditions. This review evaluates the metabolic mechanism underlying vascular aging, highlights clinical biomarkers and assessment strategies, and summarizes timely therapeutic approaches aimed at improving metabolic function.

## 1. Introduction

With the global increase in average human life expectancy, population aging has become a significant societal challenge. Advancing age substantially elevates the risk of cardiovascular disease (CVD) through cumulative structural and functional alterations in the cardiovascular system [1]. Vascular aging refers to the progressive, age-related structural and functional deterioration of blood vessels. Vascular aging disrupts glucose and lipid metabolism at cellular, organ, and hormonal levels, while glucose and lipid metabolic disorders, in turn, accelerate vascular and tissue [2, 3]. Early vascular aging (EVA) and supernormal vascular aging represent two distinct phenotypes. EVA denotes premature deterioration of arterial structure and function, particularly increased arterial stiffness, that resembles aging-related changes. In contrast, supernormal vascular aging refers to individuals with abnormally low arterial stiffness for their age and sex, representing the opposite phenotype [4].

This review examines the molecular mechanisms underlying vascular aging. The roles of endothelial cells (ECs), smooth muscle cells (SMCs), fibroblasts, inflammatory cells, and vascular stem/progenitor cells are highlighted, with particular emphasis on glucose and lipid metabolic disorders. The impact of aging on tissue and organ function is also addressed. Finally, relevant biomarkers, clinical assessment methods, and therapeutic strategies targeting vascular aging are evaluated.

## 2. Mechanism of Vascular Aging

**Fig. 1.**
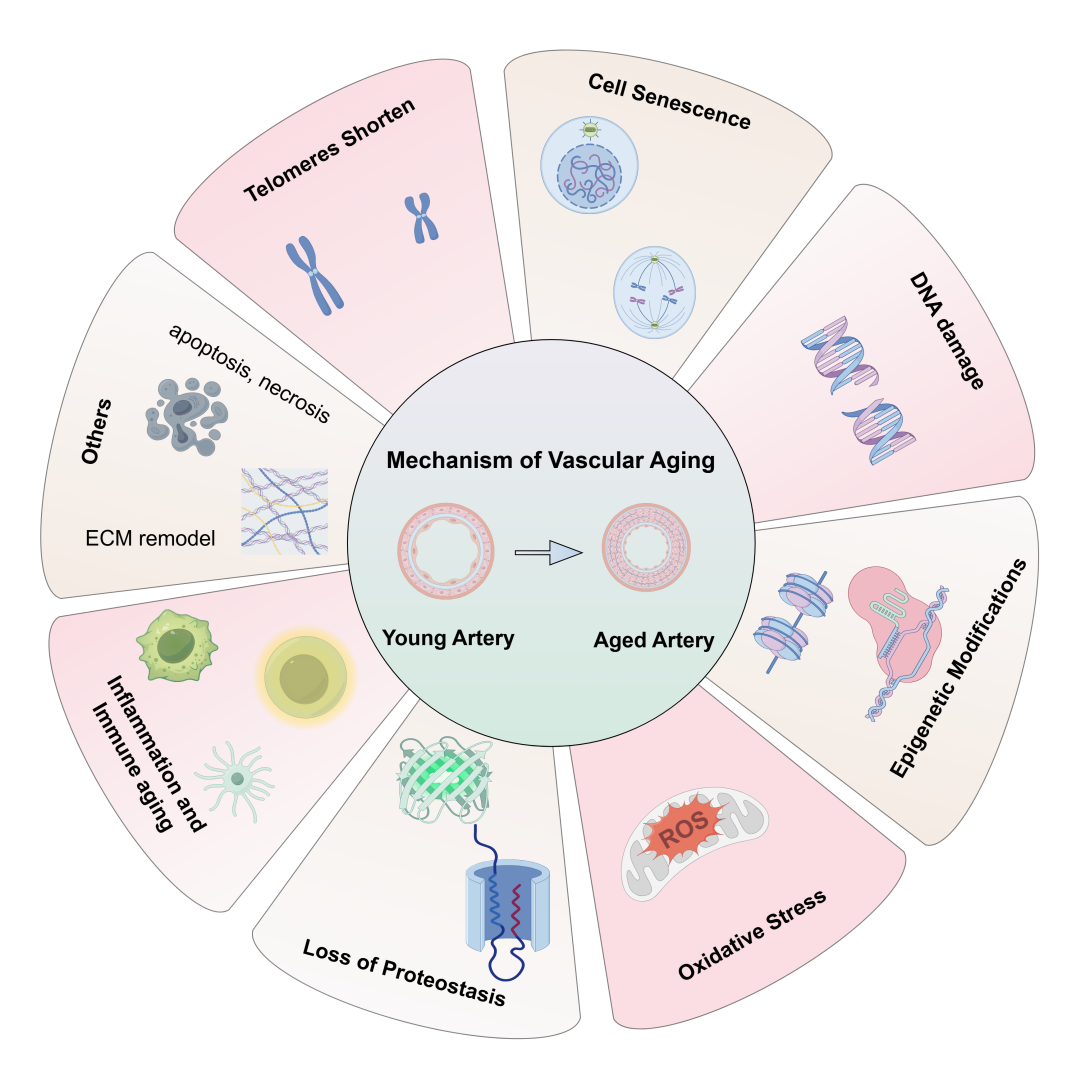
**The underline mechanism of vascular aging**. Vascular aging is also associated with telomeres shortening, cell senescence, DNA damage, epigenetic modifications, oxidative stress, loss of proteostasis, inflammation and immune aging, apoptosis and necrosis, ECM remodel. ECM, extracellular matrix; ROS, reactive oxygen species. The figure is drawn on the following website: https://www.figdraw.com.

Vascular aging encompasses progressive cellular and organismal alterations that increase the risk of dysfunction, disease, and mortality. Aging vessels are typically stiffer, thinner, and display lumen dilation along with thickening of the intima and media. Apoptosis and cellular senescence contribute to a reduced numbers of SMCs in the medial layer, and impaired intercellular communication, thereby exacerbating cellular senescence. Vascular aging is also associated with heightened oxidative stress, inflammation, and the onset of pathologies such as atherosclerosis (AS). The underlying mechanisms of vascular aging are illustrated in Fig. 1.

### 2.1 Telomeres Shortening 

Average telomere length progressively declines with age and is widely regarded as a marker of aging. Shorter telomeres have been identified in patients with AS, chronic heart failure (HF), and aortic valve stenosis. Moreover, telomere length is recognized as an independent predictor of CVD-related events [5].

### 2.2 Cell Senescence

Cellular senescence is a specific stage of irreversible proliferation arrest accompanied by persistent changes in structure and physiological function, typically induced by diverse intrinsic and extrinsic stresses during aging. It is classified into replicative senescence, oncogene-induced senescence, and stress-induced premature senescence (SIPS) [6]. Cellular senescence is characterized by an irreversible arrest at G1/S or G2 phase of the cell cycle, leading to permanent removal from the proliferative pool [7]. Sirtuins (SIRTs) are key regulators of EC senescence [8]. Inhibition of SIRT1 and SIRT6 accelerates endothelial cell senescence in culture, suggesting a positive-feedback mechanism that promotes replication driven senescence over time [9]. In ECs, SIRT1 inhibition enhances p53 activation highlighting its role in mediating endothelial senescence [10]. Reduced SIRT6 induces senescence through mechanisms upstream of p53–p21 activation, reflecting its function in telomere protection and DNA repair. Inhibition of SIRT6 in ECs results in telomere uncapping and increased nuclear DNA damage [11, 12].

### 2.3 DNA Damage

Extensive experimental evidence demonstrates that activation of the DNA damage response (DDR) by DNA double-strand breaks (DSBs) plays a central role in the development of age-related pathophysiological alterations. Mechanically, DDR initiation is characterized by the formation of DNA damage foci, a process marked by phosphorylation of histone H2AX at serine 139, at sites of DSBs. As a crucial defense system, DDR precisely regulates the cell cycle. When DNA damage exceeds the repair capacity and cannot be reversed by repair mechanisms, cells will initiate programmed cell death or enter a state of senescence. It is evident that the efficiency and outcome of DDR directly determine the final fate of cells [13].

DNA damage and the ensuing cellular senescence play a crucial role in the progression of cardiovascular disorders. DNA damage repair mechanisms have evolved to address the frequent occurrence of genomic damage, thereby preserving cellular integrity and overall health. Effective repair halts the cell cycle to prevent the propagation of damaged DNA to daughter cells [14]. Conversely, defective DNA exacerbates genomic damage and significantly accelerates senescence. Research has revealed that diverse aging-related DNA damage types initiate DNA and mitochondrial damage in ECs and modulate aging-associated endothelial inflammation through the stimulator of interferon genes/senescence-associated secretory phenotype (SASP) pathway [15].

### 2.4 Epigenetic Modifications

Emerging evidence indicates that cellular senescence is a biological process regulated dynamically by multiple epigenetic modifications. In the field of molecular biology, the core components of epigenetics include DNA methylation, histone modification, non-coding RNA regulation, and chromatin remodeling [16, 17]. Cell senescence, triggered by distinct stimuli, exhibit notable differences in gene expression profiles, phenotypes, and epigenetic signatures.

DNA methylation constitutes a fundamental epigenetic mechanism that is essential for regulating numerous biological processes [18]. Replicative senescence is characterized by a general decrease in genome-wide DNA methylation (hypomethylation) and an increase in methylation at specific sites (hypermethylation). These alterations primarily result from mislocalization, functional impairment, diminished abundance of DNA methyltransferase 1 (DNMT1) [19]. The hypermethylation phenomenon associated with the process of cellular senescence may be driven by an underlying mechanism involving HP-1-mediated targeted recruitment of DNMT1, leading to abnormal senescence-related methylation modifications. Furthermore, changes in mitochondrial DNA (mtDNA) methylation are evident in cells undergoing replicative senescence. Hypomethylation within the mtDNA noncoding region may elevate mitochondria-derived ncRNA levels, thereby affecting mitochondrial gene expression and function [19].

Histone-related epigenetics encompass histone modifications, histone variants and histone depletion. These mechanisms are involved in regulating all DNA-dependent biological activities (DNA replication, gene transcription, and DNA damage repair) [16]. Chromatin-remodeling complexes exploit ATP hydrolysis to adjust DNA-histone interactions, thereby influencing chromatin structure. Moreover, ncRNA transcripts, derived from DNA sequences comprising over 80% of the genome, are recognized as pivotal regulators of gene expression and significant contributors to cellular activities. Numerous ncRNAs display differential expression during cell senescence [20].

### 2.5 Oxidative Stress

Extensive evidence suggests that oxidative stress serve a pivotal function in vascular aging [21, 22]. In aging models, the overactivation of nicotinamide adenine dinucleotide phosphate (NADPH) oxidase and mitochondrial dysfunction disrupts the balance between the production and clearance of reactive oxygen species (ROS), which ultimately triggers endothelial dysfunction and results in arteriosclerosis. Oxidative stress also impairs vascular function by modulating key transcription factors and regulatory proteins, notably by inactivating endogenous nitric oxide (NO). With advancing age, reduced NO bioavailability may alter L-arginine and tetrahydrobiopterin availability, disrupt endothelial NO synthase (eNOS) activity, and upregulate endothelin-1 levels. Meanwhile, oxidative stress modifies the activation status of signal pathways, including nuclear factor kappa B (NF-κB) and matrix metalloproteinases (MMPs). Studies have also identified that mitochondrial ROS serves as a major contributor to the progression of vascular aging [23, 24, 25].

### 2.6 Loss of Proteostasis

Protein homeostasis maintains correct structure and function of cellular proteins. This balance is precisely regulated through the coordinated action of four core systems: synthesis, folding, trafficking, and degradation, alongside mechanisms such as the unfolded protein response, heat shock response, ubiquitin-proteasome system (UPS), and autophagy-lysosome system [17, 22]. When regulatory capacity to manage protein damage is insufficient, proteostasis is disrupted, thereby contributing to aging and associated pathologies. Heat shock protein 70 (HSP70), a component of the chaperone protein family, exhibits reduced expression in aged vascular tissues, whereas exogenous HSP70 administration extends lifespan in mouse models [26]. The UPS facilitates the activation of inflammatory responses and the accumulation of misfolded proteins in aged blood vessels. Dysregulation of adenosine monophosphate-activated protein Kinase (AMPK), phosphatidylinositol 3-kinase (PI3K) and mammalian target of rapamycin (mTOR) signaling pathways inhibits autophagy in aged blood vessels [27]. Therefore, maintaining protein homeostasis is a crucial for regulating aging.

### 2.7 Inflammation and Immune Aging

In contrast to the acute and regulated inflammation observed in young blood vessels, aged blood vessels exhibit chronic, low-grade, and persistent inflammatory activity. As aging advances, the immune microenvironment undergoes profound alterations [22, 28]. ECs and SMCs in aged blood vessels demonstrate relatively heightened NF-κB activity, which activates pro-inflammatory signaling cascades, and increases the secretion of mediators such as interleukin (IL)-6 and tumor necrosis factor-alpha (TNF-α). This process concurrently inhibits eNOS activity and upregulates the expression of endothelial adhesion molecules, including vascular cell adhesion molecule-1 (VCAM-1) and intercellular cell adhesion molecule (ICAM)-1. Macrophages shift from a repair-oriented (M2) phenotype to a pro-inflammatory (M1) phenotype. These pro-inflammatory macrophages release MMPs and damage ECs. Furthermore, senescent ECs, SMCs, and immune cells further initiate the SASP, amplifying the secretion of pro-inflammatory factors and chemokines, and accelerating vascular degeneration.

### 2.8 Other Mechanisms

Dysfunctions in nuclear factor erythroid 2-related factor 2-mediated anti-inflammatory responses and proangiogenic activity impair the functionality of senescent cells [29]. Excessive activation or suppression of pathways regulating apoptosis and necrosis accelerates the aging process. From the perspective of cellular energy and nutrient regulation, the activity of mTOR, the anti-aging SIRT enzyme family, and the AMPK signaling pathway modulates aging and lifespan. With advancing age, changes in the cytokine secretion by vascular cells, alter extracellular matrix (ECM) components composition, promoting ECM remodeling [17]. In aged blood vessels, the differentiation capacity of vascular progenitor cells into functional cells diminishes, reducing the vascular tissue’s ability to resist stress and inflammation. In summary, vascular aging is a highly complex pathophysiological process driven by multiple factors. Its primary molecular mechanisms have been outlined, yet further research is required to elucidate additional pathways and identify novel therapeutic targets for vascular aging-related diseases [30].

## 3. Biomarkers of Vascular Aging

As no pharmacological therapies currently exist to reverse vascular aging, early diagnosis of at-risk populations is essential for timely intervention. Intervention during the initial stages of disease remains the most effective approach. Identifying sensitive diagnostic biomarkers for vascular aging-related diseases is critical and urgent. Although various methods have been proposed to detect and target age-related vascular changes, their efficacy—particularly in terms of sensitivity and specificity—has yet to be established (Table 1).

**Table 1.** **Biomarkers of vascular aging**.

Telomere attrition	Shorten during aging	LTL, TRF1, TRF2
RDW	Red blood cell distribution width	Elevated RDW values, are independently associated with an increased risk of vascular aging
Sirtuins	NAD^+^-dependent deacetylases.	SIRT1, SIRT3, SIRT6, SIRT7
Cell cycle arrest	Cell cycle arrest, halting the proliferation of damaged cells	p53/p21^C⁢I⁢P⁢1^, p16^I⁢N⁢K⁢4⁢a^/RB
Epigenetic alterations	Altered DNA methylation	DNMT1, DNMT3α, DNMT3β
	Aberrant histone modifications	H3K4me3, H3K9me3
Mitochondrial biomarker	Mitochondrial dysfunction is the hallmark of aging	ROS, PGC-1, mitophagy
SASP	Senescent cells synthesize and secrete a large amount of soluble factors	Chemokines and Cytokines (MCP, CCLs, IL-6, IL-7, TNF-α, etc.)
		Inflammatory factors (TGF-β, IFN-γ, MFG-8, etc.)
		Growth factors (EGF, VEGF, SCF, etc.)
		Others (ICAM, MMPs, etc.)

RDW, red blood cell distribution width; SASP, senescence-associated secretory phenotype; LTL, leukocyte telomere length; CCLs, c-c motif chemokine ligands; TGF, transforming growth factor; IL, interleukin; TNF, tumor necrosis factor; IFN, interferon; MFG, milk fat globule-epidermal growth factor; EGF, epidermal growth factor; VEGF, vascular endothelial growth factor; SCF, stem cell factor; ICAM, intercellular cell adhesion molecule; MMPs, matrix metalloproteinases; SIRT, sirtuin; PGC-1, peroxisome-proliferator-activated receptor-γ co-activator-1; DNMT, DNA methyltransferase.

### 3.1 Telomere Attrition 

Telomere length is a recognized biomarker for aging. Telomeric repeat-binding factors (TRF1 and TRF2) ensure that telomeres maintain genomic stability during cell division and aging. Meta-analyses have indicated that short telomeres are strongly associated with atherosclerotic cardiovascular disease (ASCVD) mortality, particularly in the younger age individuals. Recent research has demonstrated that blood leukocyte telomere length (LTL) acts as a mitotic timer for determining human biological age and is a molecular indicator for EVA [31]. Studies indicate that themean relative LTL declines with physiological aging and is linked to premature stable coronary artery disease (CAD), and accelerated vascular aging [32, 33].

### 3.2 Cell Cycle Arrest

The cell cycle orchestrates cellular proliferation and division, which includes the G1, S, G2, and M phases. A hallmark of cellular senescence is cell cycle arrest, with senescent cells predominantly maintained in a stable arrest in G1 or G2 phase, thereby losing their proliferative capacity. Research has demonstrated that p53/p21^C⁢I⁢P⁢1^ and p16^I⁢N⁢K⁢4⁢a^/RB pathways are key signaling cascades that regulate this cell cycle arrest. In senescent vascular SMCs (VSMCs) and ECs, consistent upregulation of p53/p21^C⁢I⁢P⁢1^ and p16^I⁢N⁢K⁢4^ pathways is observed [17].

### 3.3 Red Blood Cell Distribution Width (RDW)

RDW is a standard parameter in complete blood countanalysis, quantifying the degree of erythrocyte anisocytosis and reflecting variations in red blood cell size and morphology. Recent clinical evidence has linked RDW values tothe vascular aging and the incidence and prognosis of abdominal aortic aneurysm (AAA) [34].

### 3.4 Epigenetic Alterations

It is well established that epigenetic modifications constitute a key hallmark of aging. DNA methyltransferases (DNMTs) (DNMT1, DNMT3A, DNMT3B) are essential regulators of DNA methylation and influence AS progression [35]. Histone modifications in senescent cells have been extensively characterized, particularly methylation (H3K4me3 and H3K9me3) and acetylation. Aberrent acetylation patterns in hutchinson-gilford progeria syndrome (HGPS) VSMC replication fork complexes reduce H4K16 acetylation. Primary donor-derived coronary artery VSMCs from aged individuals exhibit similar defects to those of HGPS VSMCs, including loss of H4K16 acetylation [36].

### 3.5 Mitochondrial Malfunction

Mitochondria perform diverse biological functions, possess their own genetic material–mtDNA–that is independent of the nuclear genome, and serve as the primary site of ROS production. ROS generation and mtDNA mutations are closely associated with age-related diseases. A study demonstrated that ablation of peroxisome proliferator-activated receptor γ coactivator-1α accelerates vascular aging and AS, accompanied by mitochondrial dysfunction, telomere shortening, and DNA damage [37].

### 3.6 SIRTs

Emerging evidence suggests that SIRTs serve a vital function in vascular aging by repairing DNA damage, reducing oxidative stress, and mitigating inflammation. Decline in SIRT1 levels during early adulthood increases the risk of CVD through microvascular dysfunction [38, 39]. Activation and overexpression of SIRT2 stabilize atherosclerotic plaques by regulating lipid metabolism and gluconeogenesis [40, 41]. A study conducted in mice lacking SIRT3 and low-density lipoprotein (LDL) revealed that oxidative stress and adverse lipid profiles were significantly elevated in the absence of SIRT3 [42]. The SIRT6 contributes to telomere maintenance and the prevention of aging, as evidenced by the accelerated senescence observed in SIRT6-deficient mice [43]. Research demonstrates that SIRT7 provides protection against cardiorenal disease, and remains the least characterized SIRT [44].

### 3.7 SASP

Senescent cells secrete large quantities of soluble molecules, including cytokines, chemokines, inflammatory mediators, growth factors, and other bioactive factors. The SASP constitutes a highly complex signaling network, with key components such as matrix cell crotein (MCP), c-c motif chemokine ligand (CCL)s, IL-6, IL-7, TNF-α, transforming growth factor (TGF)-β, Interferon (IFN)-γ, milk fat globule-epidermal growth factor (MFG)-8, epidermal growth factor (EGF), vascular endothelial growth factor (VEGF), stem cell factor (SCF), ICAM, and MMPs [45]. Multiple studies have reported a significant increase in SASP secretion from senescent ECs and SMCs, aggravating the progression of age-related vascular disorders [17, 30].

## 4. Disorder of Glucose and Lipid Metabolism Promotes the Progression of Vascular Aging

### 4.1 Disorder of Glucose and Lipid in Aging ECs

EC dysregulation contributes endothelial dysfunction and aortic disorders, compromising the protective barrier of vascular intima. Similar to other mammalian cells, ECs exhibit reduced proliferative capacity with age. Endothelial triggers a cascade of events that lead to cardiovascular and other EC dysfunction-related diseases. Senescent ECs demonstrate flattened and enlarged morphology, diminished DNA replication, telomere shortening, increased senescence-associated β-galactosidase (SA-β-gal) activity, along with cell cycle arrest.

Glycolysis contributes approximately 80% of ATP production in ECs. Reduced glycolysis decreases EC proliferation, migration, whereas stimulation of ECs with VEGF and fibroblast growth facto (FGF)-2 enhances glycolytic activity [46]. Deletion of cystathionine γ-lyase in murine ECs activates p53, induces endothelial senescence, and halts arterial repair [47]. Aging-related arterial dysfunction involves various factors, it including the maintenance of shear-stress-induced NO production via glycolysis-dependent purinergic signaling to eNOS. Additionally, purinergic 2Y1-receptor (P2Y1-R) activation promotes EC autophagy, which declines with age [48]. Hyperhomocysteinemia (HHcy) represents a distinct metabolic syndrome often associated with defects in methionine-cysteine metabolism defects and nutritional deficiencies. Severe HHcy contributes to vascular pathologies through EC death. Sub-lethal Hcy elevation impairs endothelial proliferation by inhibiting mitochondrial respiration, inducing compensatory glycolysis to sustain ATP production and energy homeostasis [49].

**Fig. 2.**
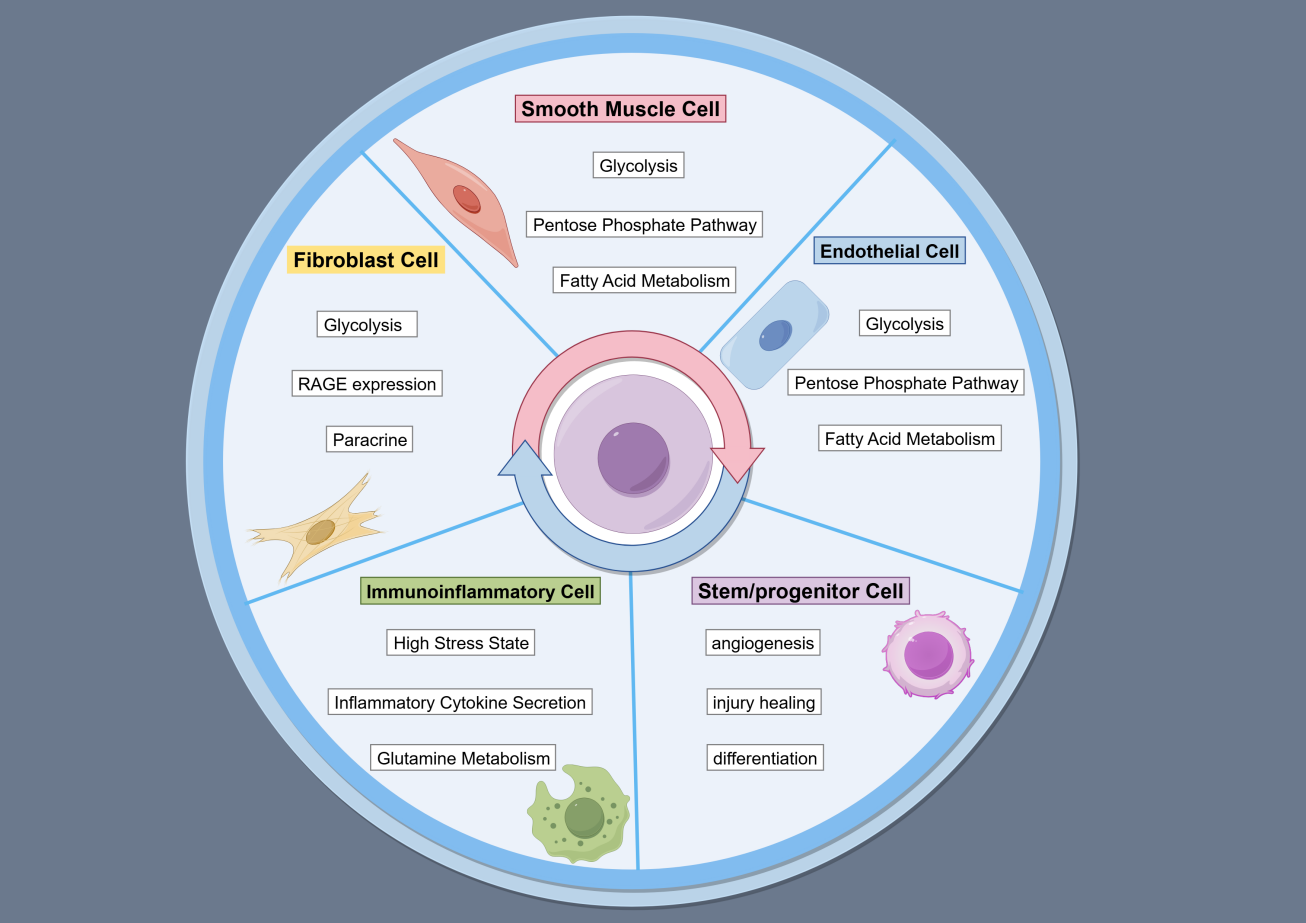
**Disorder of glucose and lipid in age-associated vascular cells**. The figure is drawn on the following website: https://www.figdraw.com.

EC function is modulated by fatty acid metabolism, as carnitine palmitoyltransferase 1 (Cpt1) deficiency reduces EC proliferation and increases endothelial permeability [44]. By regulating acetyl-coenzyme A (acetyl-CoA) metabolism, fatty acid metabolism controls endothelial senescence. Inhibition of ATP citrate lyase by NDI-091143 inhibits decreases acetyl-CoA production in HUVECs, accelerating their senescence, whereas acetyl-CoA supplementation delays H_2_O_2_-induced senescence in HUVECs [50]. Cylindromatosis (CYLD) expression in ECs and macrophages declines with age, enhancing monocyte adhesion to the endothelium and promoting foam cell formation, thereby contributing contributes to age-related atherogenesis (Fig. 2) [51].

### 4.2 Disorder of Glucose and Lipid in Aging SMCs

SMCs are essential for the physiological functions of the vascular wall. VSMCs exhibit high plasticity and advances in single-cell sequencing and cell-lineage tracing have linked them to diverse phenotypes in vascular aging, AS, aortic aneurysm, and relatedconditions [51, 52]. Compared to patients with Alzheimer’s disease, nonagenarians exhibit enhanced oxidative stress, altered gluconeogenesis-glycolysis pathways, and maintained SMC vasodilation effects [52]. In neointimal VSMCs of human stenotic carotid arteries and wire-injured mouse carotid arteries, hypoxia-inducible factor-1αand its target 6-phosphofructo-2-kinase/fructose-2,6-bisphosphatase (PFKFB3) drive phenotypic switching through FASN-mediated lipid metabolism and PFKFB3-mediated glycolysis [53]. The nuclear receptor subfamily 4 group A member 3 protein regulates AS progression induced by apolipoprotein A-IV [54].

AS can be triggered by oxidized lipids and their metabolites, which, in addition to exerting cytotoxic and chemotactic effects, induce macrophage apoptosis [55]. Oleic acid stimulates SMC proliferation and activates free fatty acid receptor 1 (FFAR1) and PI3K/AKT signaling. Metabolomic analysis revealed a marked reduction of leucine aged mice aortas. Through Sirt1-mediated Foxo1 deacetylation, leucine modulates VSMC phenotypes, ameliorating aging-induced vascular remodelling in mice [56]. Furthermore, platelet-derived growth factor subunit B (PDGF-B) treatment of VSMCs increases FAO and decreases glycolysis, the latter of which contrasts with other reports (Fig. 2) [57].

### 4.3 Disorder of Glucose and Lipid in Vascular Fibroblast Cells

The healthy arterial wall exhibits a typical three-layered structure, consisting of the intima, media, and adventitia. In contrast to the intima and media, the adventitia harbors diverse cell types, including ECs of the vasa vasorum, immune cells, mesenchymal cells, and vascular progenitor cells. Myofibroblasts, the most abundant adventit cells in the adventitia, migrate to the intima in response to injury or environmental stress. Their invasion and elevated ECM protein production drive arterial remodeling, intimal hyperplasia, and arterial stenosis.

High glucose upregulates glycolytic enzymes and transglutaminase 2 (TG2), stimulating glycolysis in fibroblasts. TG2 inhibition suppresses glucose-induced fibroblast proliferation and fibrogenesis [58]. Most proteins with differential expression profiles in human adventitial (hAdv) cells treated with conditioned medium (CM) from human aortic endothelial cells (HAEC) are associated with lipoprotein metabolism, mitophagy, and ferroptosis [59]. Fibroblasts secrete chemokines, cytokines, and glycolytic metabolites that recruit, retain, and activate naïve macrophages (Mϕ) into a pro-inflammatory/pro-remodeling phenotype. Fibroblast-activated macrophages undergo aerobic glycolysis and exhibit elevated arginase 1, Vegfa, and I1lb levels, dependent on hypoxia-inducible factor 1 and signal transducer and activator of transcription 3 (STAT3) signaling [60]. During diabetic hyperglycemia, the aorta undergoes mineral dysregulation, leading to an increased cellular responsiveness to calcification-inducing chemical messengers. Advanced glycation end products (AGEs)/receptor for advanced glycation end products (RAGEs) signaling mediates diabetes-induced vascular calcification, with diabetic adventitial fibroblasts (AFBs) exhibiting reduced RAGE expression following calcification and AGE treatments [61]. Media from diabetic preconditioned VSMC reduces H α-smooth muscle actin. Whereas VSMCs preconditioned with non-diabetic calcified plus AGE exhibit higher levels of superoxide dismutase-2 levels than diabetic VSMCs under the same treatment (Fig. 2) [62].

### 4.4 Disorder of Glucose and Lipid in Vascular Immunoinflammatory Cells

Prolonged hyperglycemia places macrophages under heightened stress, provoking exaggerated responses external stimuli. Consequently, excessive inflammatory factors including TNF, IL-6, and CCL2, promote M1 polarization [63]. As reported by Matsuura *et al*. [64], diabetes decreases lyceraldehyde-3-phosphate dehydrogenase (GAPDH) and glucose transporter 1 (GLUT1) gexpression in macrophages, as a result diminishing glucose uptake and glycolytic activity. M1 macrophages secrete inflammatory cytokines, such as IL-1, contributing to insulin resistance in the musculoskeletal tissue, adipose tissue (AT) and liver, and also inducing pancreatic dysfunction [65, 66, 67].

Patient with type 2 diabetes mellitus (T2DM) exhibit impaired neutrophil functions, including ROS production, bactericidal activity and neutrophil extracellular trap (NET) formation [68]. Thimmappa *et al*. [69] found that in the presence of high glucose levels, neutrophil functions in both T2DM and healthy donors compete for NADPH. As a result, high glucose treatment increases cytosolic ROS production, while insufficient NADPH limits lipopolysaccharide-induced NET production. NADPH supplementation and aldose reductase inhibition-targeting the enzyme that converts glucose to sorbitol using NADPH oxidase-restores NET formation under high glucose conditions and reduces cytosolic ROS, and spontaneous NET release induced by high glucose (Fig. 2) [70, 71].

### 4.5 Disorder of Glucose and Lipid in Vascular Vtem/Progenitor Cells

Endothelial progenitor cells (EPCs), which are bone marrow-derived CD34^+^ cells, serve as key biomarkers for CVD risk and play a critical role in prevention and therapy. Preclinical and clinical studies have demonstrated that dipeptidyl peptidase-4 (DPP4) inhibitors prevent vascular disease by controlling EPC number and function, highlighting a correlation between cardiovascular risk and disease [72]. A study investigated whether age affects EPC function and its relationship with systemic inflammation in 58 patients with HF exhibiting mildly reduced ejection fraction. The results indicated that CD34^+^ cells from older subjects (>50 years) contained 3.5 times more glycogen and exhibited greater heterogeneity than those from younger subjects (<35 years) [73]. Additionally, older subjects demonstrated a markedly increased glycolysis in hematopoietic progenitor cells [74]. Mesenchymal stem cells (MSCs), as multipotent stromal cells, differentiate into multiple lineages and exhibit immunosuppressive properties. *In vitro*, MSC-derived small extracellular vesicles (MSC-sEV) mitigated senescence biomarkers and SASPs, thereby restoring angiogenesis, migration, and other physiological functions in senescent EC. MSC-sEV facilitated wound closure and angiogenesis in mice with senescent ECs, including those in T2DM wound healing models. MicroRNA (miRNA) microarray analysis revealed increased miR-146a levels in MSC-sEV, which were further elevated in ECs after MSC-sEV treatment [75]; inhibition of miR-146a abolished these pro-senescence effects. Another study, reported that bone marrow-derived MSCs from aged mice exhibited significantly reduced oxidative phosphorylation and glycolytic activity (Fig. 2) [76].

## 5. Effects of Vascular Aging on Glucose and Lipid Metabolism

### 5.1 Liver

In the liver, glycolysis, glucose uptake, and gluconeogenesis regulate key metabolic pathways, including carbohydrate metabolism. Liver metabolism declines with age due to three primary factors: reduced metabolic capacity resulting from a smaller liver size or decreased enzyme levels, impaired blood flow, and decreased transfer of metabolites and molecules between sinusoids and hepatocytes. Liver sinusoidal ECs (LSECs) undergo morphological and functional alterations during aging and disease, notably loss of fenestrations, termed “defenestration” which is often linked to basement membrane formation. Aging in mice and humans is associated with a marked reduction in LSEC porosity driven by increased cross-sectional thickness. These age-related histological changes coincide with altered expression of numerous vascular-related proteins, including alpha-laminin, willebrand factor, caveolin-1, ICAM-1, and multiple collagen isoforms.

In older adults, chylomicron remnant clearance is impaired accompanied by postprandial hypertriglyceridemia. Multiple indicator dilution method in perfused rat livers revealed near-complete cessation of lipoprotein transfers across the LSECs in aged livers [77], suggesting a potential mechanism for age-related dyslipidemia and hyperlipidemia, which may contribute to vascular disease. Aging is also associated with increased risk of diabetes and insulin resistance. Older adults exhibit impaired insulin transfer across LSEC confirmed by multiple indicator dilution techniques in perfused livers. In older rats, hepatic insulin distribution was significantly reduced, largely restricted to the vascular space. Whole animal studies have demonstrated age-related reductions in hepatic insulin and glucose uptake, accompanied by decreased activation of the insulin pathway. Impaired hepatic insulin action was linked to systemic deficits in insulin sensitivity and glycolysis, as evidenced by glucose tolerance and homeostasis model assessment of insulin resistance (HOMA-IR) measurements. These findings suggest that fenestrations affect hepatic insulin uptake. In contrast, mice lacking PDGF-B exhibited increased fenestrations, enhanced insulin sensitivity and insulin clearance, and markedly lower circulating insulin levels [78, 79].

### 5.2 Skeletal Muscle

The vascular endothelium serves a pivotal function in regulating skeletal muscle metabolism. In a murine obesity model, p53 levels in the vascular endothelium were elevated [80]. Genetic depletion of endothelial p53 reduced visceral and subcutaneous fat accumulation and improved insulin resistance. In skeletal muscle, eNOS activates peroxisome proliferator-activated receptor-coactivator-1, whereas p53 inhibits eNOS activity. Endothelial p53 depletion enhanced skeletal muscle glucose uptake through upregulation of GLUT1. These findings indicate that suppressing vascular aging promotes mitochondrial biogenesis in skeletal muscle, thereby improving metabolic health [81].

### 5.3 AT

AT in several forms, including white, brown, and beige fat. Brown AT (BAT) was initially considered a thermogenic organ, abundant in newborn babies and rodents, subsequent research has demonstrated its presence in adults. Beyond thermogenesis, BAT regulates systemic metabolism. The mechanisms underlying the decline of BAT activity in adults with obesity and aging remain incompletely understood. Metabolic stress reduces VEGF-A expression in brown adipocytes via fatty acid accumulation, a key proangiogenic molecule. Consequently, BAT undergo escapillary rarefaction and hypoxia to a greater extent than in white AT, leading to “whitening” which is associated with decreased adrenergic signaling, lipid droplet accumulation, and mitochondrial dysfunction [82, 83].

Transcriptome analysis of senescent vascular ECs identified glycoprotein non-metastatic melanoma protein B (GPNMB), a molecule and seno-antigen. Genetic ablation of Gpnmb-positive cells in high-fat dietfed mice reduced AT senescence, ameliorated metabolic abnormalities, and attenuated AS in apolipoprotein E-deficient mice. Cellular senescence in AT contributes to metabolic dysfunction, as indicated by improved insulin resistance following p53 inhibition. Aging AT-associated inflammation and lipid redistribution promot emetabolic disturbances, including insulin resistance, impaired glucose tolerance, and diabetes [72]. EC-specific Tert knockout in EC accelerated telomere attrition in AT EC, accompanied by reduced mitochondrial content and function, thereby increasing reliance on glycolysis [84].

## 6. Advanced Multi-omics Application for Vascular Aging

Recent advances in molecular biology, genetics, and bioinformatics have transformed aging research. The identification of key genes, proteins, molecules, and signaling pathways has enhanced our understanding of aging mechanisms. Systems biology and integrative multi-omics analyses are currently central approaches inaging research, aiming to elucidate the complexity of aging.

### 6.1 Genomics

Genomics investigates an organism’s complete DNA sequence, encompassing both coding and noncoding regions. Techniques such as whole-genome sequencing, whole-exome sequencing, and gene chips enable the identification of genetic variations, epigenetic regulation, gene mapping, and associations with genetic diseases. Studies have demonstrated that specific single-nucleotide polymorphisms in the *forkhead box O3 (FOXO3)* gene, such as *rs2802292*, correlate with a significantly slower rate of vascular endothelial function [85]. These genetic markers provide direct evidence for screening high-risk populations for CVD-related aging. Furthermore, epigenomic analyses can more accurately predict the degree of vascular stiffness and the rate of decline in diastolic function. Methylation levels of *ELOVL2* and *CCDC102B* genes in coronary artery ECs correlate positively with atherosclerotic plaque burden [86, 87].

However, genomic sequences remain largely stable throughout an individual’s lifetime, limiting their capacity to capture the “dynamic changes” associated with vascular aging. Moreover, the polygenic nature of aging may reduce the reliability of identifying causal associations. However, genomic sequences remain stable throughout an individual’s lifetime, making them unable to explain the “dynamic changes” occurring during vascular aging. Additionally, the aging process is determined by multiple genes, which may limit the reliability of identifying causal associations.

### 6.2 Transcriptomics

Transcriptomics, particularly single-cell RNA sequencing (scRNA-seq), examines spatiotemporal variations in gene expression within cardiovascular tissues, revealing cellular subtype imbalances and pathway abnormalities. In aging models of AS, scRNA-seq has demonstrated that the proportion of anti-inflammatory vascular ECs decreases from 35% in young mice to 12% in aged mice, whereas the pro-inflammatory ECs increase from 10% to 40% [88]. Spatial transcriptomics enables the analysis of gene expression distribution, uncovering transcriptional abnormalities in local microenvironments. Moreover, temporal transcriptome sequencing allows dynamic tracking of critical transition points during vascular aging [89].

Nevertheless, correlations between messenger RNA (mRNA) expression and protein activity are typically in the range of 0.3–0.5. In cardiovascular aging, abnormalities in translational regulation can lead to increased mRNA expression without corresponding changes in protein levels. Additionally, scRNA-seq may induce cell damage or transcript degradation, and rare cell subtypes are prone to being overlooked during this process.

### 6.3 Proteomics

Proteomics offers a powerful strategy for identifying diagnostic and prognostic markers as well as elucidating pathophysiological mechanisms. Its methodologies are widely applied to investigate alterations in protein abundance associated with disease states, therapeutic responses, and age-related changes in plasma proteomes [90, 91]. Proteomics provides direct insights into the “functional abnormalities” underlying vascular aging. The high stability of proteins enhances their suitability as circulating biomarkers in fluids such as blood or cerebrospinal fluid.

Nevertheless, the precision of protein detection is influenced by protein concentration, and proteins demonstrate tissue-specific variability. Accordingly, diverse analytical approaches are required to obtain and examine samples from target tissues, such as vascular endothelium or myocardium.

### 6.4 Metabolomics

Metabolomics examines alterations in small-molecule metabolites within cardiovascular tissues and body fluids. It provides direct evidence for glycolysis and lipid accumulation in cardiovascular aging. Spatial metabolomics has proven to be an effective tool for detecting metabolic states of cells and their heterogeneity [92]. Metabolites are direct products of cellular function, and clinically validated detection technologies, such as nuclear magnetic resonance and liquid chromatography-tandem mass spectrometry, provide broad applicability, establishing metabolomics as an ideal tool for screening indicators [93].

Nonetheless, metabolite levels remain susceptible to external factors, including diet, exercise, pharmacological treatments, and circadian rhythms. Consequently, the significance of metabolite data is established primarily when corroborated by intervention studies.

The complexity of vascular aging indicates that no single omics approach can fully elucidate its mechanisms [94]. Future investigations should prioritize integrated multi-omics approaches, longitudinal monitoring, and cross-population validation to advance CVD prevention and treatment strategies targeting vascular aging with precision.

## 7. Clinical Application

### 7.1 Clinic Assessment of Vascular Aging

Pulse wave velocity (PWV) quantifies the speed of the blood pressure pulse through vessels and serves as a standard indicator of arterial stiffness [95, 96]. In clinical application, the calculation of PWV involves dividing the distance between two measurement sites by the pulse travel time, with the resulting value representing PWV. Several methods have been developed to evaluate PWV: (a) Carotid-femoral PWV (cf-PWV) is the predominant method for assessing arterial stiffness. The carotid-femoral path length is typically estimated by multiplying the distance between the two sites by 0.8 [96, 97]. (b) Brachial-ankle PWV (ba-PWV) is widely used as an alternative approach, measuring the pulse wave transit between the brachial and ankle arteries. The distance between the two measuring sites is determined using linear regression with body weight [96, 98]. (c) The cardio-ankle vascular index (CAVI) is a parameter derived from PWV, primarily associated with the stiffness and compliance of the descending aorta [99]. It is used to evaluate vascular stiffness and as an indicator of arteriosclerosis. Carotid intima-media thickness (CIMT) is the distance between the lumen and adventitial surfaces of the carotid arteries, which grows steadily with age and independently predicts cardiovascular events [100]. The Flow-mediated dilation (FMD) is currently recognized as the reference standard for evaluating vascular endothelial function and is widely applied in clinical application and nutritional investigation [101]. The procedure utilizes a two-dimensional ultrasound probe to measure brachial artery diameter before and after arterial occlusion. The ankle-brachial index is a simple and practical tool for assessing peripheral vascular damage in advanced AS, calculated as the ratio of systolic blood pressure measured at the ankle to that at the brachial artery [102]. The Framingham Vascular Age Calculator is a tool used to assess vascular age, developed based on the Framingham Cohort Study [103]. It generates a vascular age value by collecting an individual’s data such as age, gender, smoking status, blood pressure, cholesterol, and glucose. Computed tomography (CT) angiography is widely used to diagnose vascular structural abnormalities. Various noninvasive approaches such as CT-derived fractional flow reserve, are applied to estimate and predict blood flow distribution following coronary stenting. Quantitative magnetic resonance imaging enables noninvasive evaluation of vascular hyperemia, impaired by endothelial and structural dysfunctions. Techniques include blood oxygenation-level dependent imaging for blood flow and capillary oxygen content, arterial spin labeling for regional perfusion, and phase contrast for arterial flow waveforms, macrovascular blood flow rate, and velocity (Table 2) [104, 105].

**Table 2.** **Clinic assessment of vascular aging**.

Clinic assessment	PWV	Pulse wave velocity	measures the speed of the blood pressure pulse through vessels
	cf-PWV	Carotid-femoral PWV	the carotid-femoral path length can be calculated by multiplying the distance between the two points by 0.8
	ba-PWV	brachial-ankle PWV	measuring the pulse wave transit between the brachial and ankle arteries.
	CAVI	Cardio-ankle vascular index	derived from PWV
	ABI	Ankle brachial index	comparing the systolic blood pressure measured at the ankle with the systolic blood pressure measured at the brachial artery
	CIMT	Carotid intima-media thickness	the distance between lumen and adventitia surfaces
	FMD	Flow-mediated dilation	measure the diameter of the brachial artery before and after arterial occlusion
	Vascular aging scores	Framingham vascular age calculator	generate a vascular age value by collecting an individual’s data such as age, gender, smoking status, blood pressure, cholesterol, and glucose
	CTA	the most commonly used clinical techniques for diagnosing diseases associated with vascular structure variation	
	MRI	measuring Vascular hyperemia, impaired by endothelial and structural dysfunctions	

### 7.2 Clinic Research 

Age-related vascular changes contribute to vascular cognitive impairment. Multiple linear regression analysis was used to evaluate the effects of metabolic syndrome (MetS), baPWV, and their interaction on cognitive performance. Individuals with MetS and elevated ba-PWV demonstrated the greatest impairment [106]. A cross-sectional descriptive study of 500 participants analyzed the relationship between addictions and obesity, including physical activity, body fat distribution, arterial stiffness, sedentary time, gender differences and cognitive function. Coronary artery stiffness and vascular aging progress gradually from an early age and contribute to morbidity and mortality in patients with CVD [107]. Untargeted plasma metabolomics were measured using liquid chromatography mass spectrometry in 6865 individuals across two Swedish cohorts. The objective was to predict aortic stiffness through direct assessment with cf-PWV and indirect assessment via the augmentation index (AIx@75). A 23-year longitudinal study demonstrated that aortic stiffness predicted by metabolites is statistically significantly associated with the incidence of newly diagnosed CVD, cardiovascular mortality [108]. The Kailuan prospective cohort study further investigated the relationship between adverse pregnancy outcomes (APOs) and vascular aging, assessed through elevated PWV in young women, participants underwent postpartum baPWV measurements. Multivariable logistic regression revealed an association between APOs and elevated PWV. APOs are identified as risk factors for vascular aging in young women, although the risk decreases with age. Therefore, Ba-PWV is an essential indicator for preventive cardiovascular risk management in this population [109].

### 7.3 Delay Vascular Aging

The core goal of vascular aging treatment is to delay the structural and functional deterioration of vascular tissues and reduce the risk of related CAD. Fundamental treatments include lifestyle interventions and pharmacologic treatments. Due to the numerous researches on mechanisms of vascular aging, new anti-aging therapies include senolytics, senomorphics, and anti-aging vaccines [110, 111]. In this review, we explore therapeutic strategies for improving glucose and lipid metabolism to delay aging.

#### 7.3.1 Exercise Intervention

Extensive research demonstrates that exercise enhances health span during aging. Experimental evidence indicates that exercise prevents senescence and suppresses components of the pro-inflammatory SASP in the sera and hearts of aged mice. Exercise also mitigates diet-induced senescence and SASP activation in mouse adipose and liver tissues, suggesting that its greatest benefits are observed in subjects at risk of metabolic disease rather than in those already healthy [112]. In humans, regular physical activity is associated with reduced markers of endothelial and leukocyte cell senescence [113].

#### 7.3.2 Dietary Control 

Dietary habits exert profound effects on vascular function. Multiple studies have confirmed that mediterranean diet (MedDiet) can reduce the morbidity and mortality of cardiovascular diseases, as well as T2DM. MedDiet is reported to improve endothelial function and delay AS progress [114]. After 8 weeks of a 1000 kcal/day caloric restriction, and rose further to 12.46% over the subsequent 44 weeks [115]. An 8-week low-fat dairy decreased cf-PWV in participants, with hypercholesterolemia, with the reductions strongly correlating with decreased plasma c-reactive protein (CRP) levels [116]. A high-fiber diet was associated with a reduced risk of Cardio-cerebral vascular diseases (CCVDs), decreased cIMT, restoration of endothelial function in MetS patients, and prevention of endothelial dysfunction induced by fat-rich meals. High sodium intake negatively influences vascular function. A comparative study reported that an 8-week, intermittent fasting regimen (2 fasting days per week) improved circulating markers of endothelial function-including total nitrate, asymmetric dimethylarginine, and VCAM in patients with MetS [117].

#### 7.3.3 Treatments for Diabetes 

Hyperglycemia, a hallmark of diabetes and impaired glucose metabolism, accelerates vascular aging by promoting AGE formation and EC senescence, thereby exacerbating arterial stiffness and endothelial dysfunction [118]. Patients with newly diagnosed diabetes exhibit approximately 14% lower FMD compared with age-matched healthy controls. Consequently, anti-hyperglycemic therapy is essential for preventing vascular aging. Metformin remains the typically first-line pharmacologic treatments for T2DM due to its insulin-sensitizing effects and significant improvement of age-related vascular function. The REMOVAL trial demonstrated a 0.012 mm/year reduction in the age-related cIMT progression in patients with diabetes treated with metformin for 5 years [119]. Other glucose-lowering drugs, including sodium-glucose co-transporter-2 (SGLT2) inhibitors, also improve age-related vascular phenotypes similar to metformin [120]. SGLT2 inhibitors block glucose reabsorption within the proximal convoluted tubules, thereby promoting glucose excretion. Empagliflozin demonstrates superior efficacy in reducing cf-PWV in patients with type 1 diabetes. Endothelial function improves more in patients with T2DM receiving combined insulin and metformin therapy than in those treated with metformin alone [121].

#### 7.3.4 Lipid-lowering Agents 

**Fig. 3.**
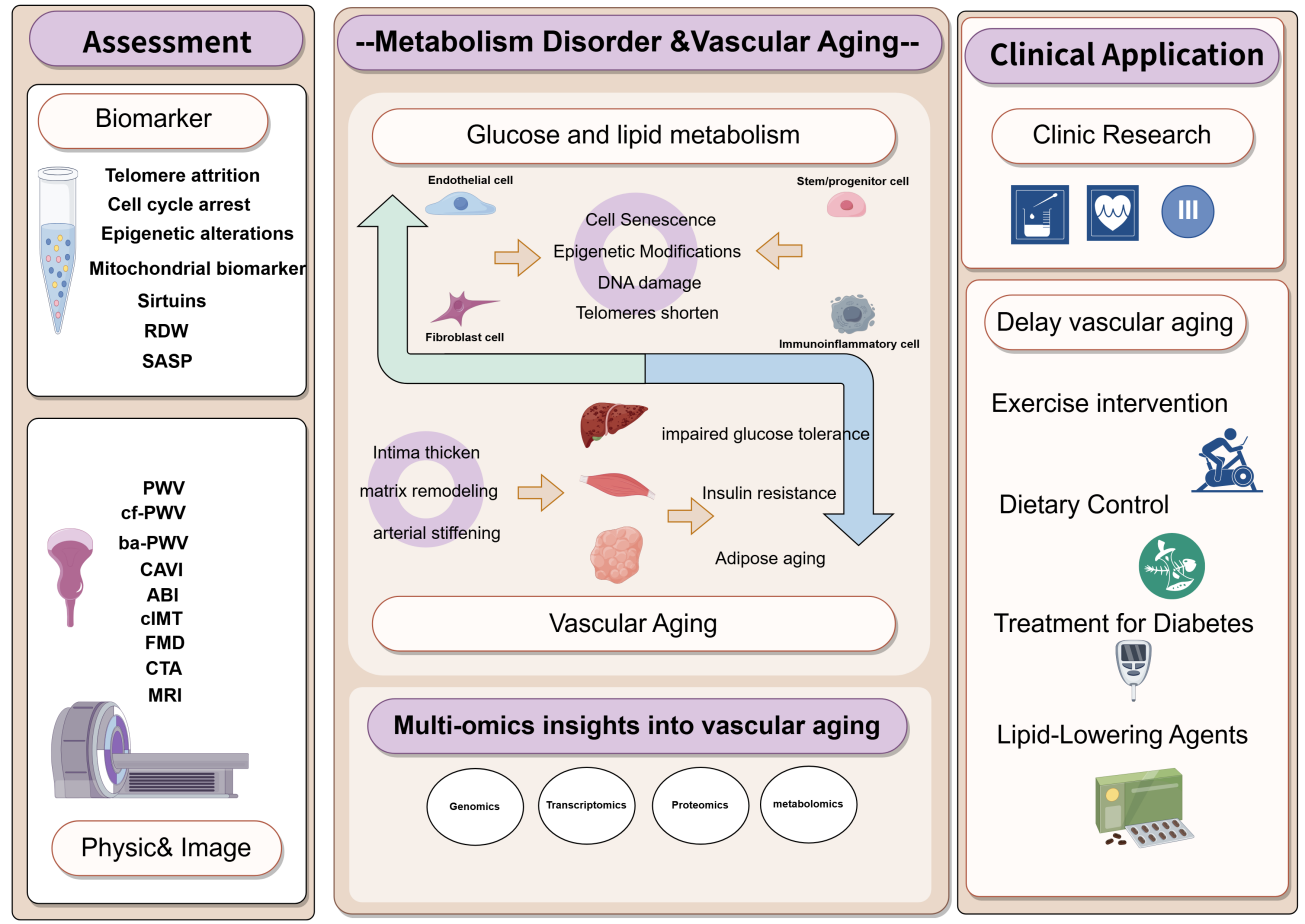
**The interaction between glyco-lipid metabolism and vascular aging, its clinical application**. Clinic assessment of vascular aging include biomarker and physical& image examination. More clinical research is needed to delay vascular aging. PWV, pulse wave velocity; cf-PWV, carotid-femoral PWV; ba-PWV, Brachial-ankle PWV; CAVI, cardio-ankle vascular index; CIMT, carotid intima-media thickness; FMD, flow-mediated dilation; ABI, ankle brachial index; CTA, computed tomography angiography; MRI, magnetic resonance imaging. The figure is drawn on the following website: https://www.figdraw.com.

Hyperlipidemia impairs ECs and EPCs, reducing their ability to repair vascular injury [122]. Patients with familial hypercholesterolemia exhibit increased CIMT and cf-PWV compared with healthy individuals, reflecting premature vascular aging in hyperlipidemia [123]. Statins can inhibit vascular injury induced by chronic inflammation and oxidative stress, and improve endothelial function in patients with AS [124]. Evidence suggests that statin therapy exerts pleiotropic effects, that enhance endothelial function, even before the full lipid-lowering effects are achieved. However, recent research on the co-administration of simvastatin and ezetimibe to attain greater reductions in LDL-C indicates that improvements in endothelial function are more closely associated with lipid reduction. A study in individuals with chronic kidney disease reported that statins may slow the progression of arterial stiffness [125]. A longitudinal study further demonstrated that 2 years of atorvastatin treatment reduced aortic dimension in hypercholesterolemic patients. Moreover, research indicates that low-dose simvastatin decreased LDL-C levels by 25% without influencing CIMT, whereas high-dose atorvastatin significantly reduced LDL-C by 45% and reversed age-related CIMT progression [72]. Collectively, these findings suggest that more intensive and prolonged statin treatments are required to achieve structural improvements in arteries (Fig. 3) [124].

## 8. Conclusion and Future Perspectives

Age-related alterations in glucose and lipid metabolism are closely linked to vascular aging. Several underlying pathophysiological mechanisms have been elucidated, including insulin resistance and a chronic dysregulation of glucose and lipid metabolism, which impair AT homeostasis. This review summarizes the mechanisms and biomarkers of vascular aging, emphasizing the influence of glucose and lipid metabolism on vascular cellular senescence (ECs, SMCs, adventitial fibrocytes, immune cells and stem/progenitor cells), as well as the reciprocal effects of vascular aging on tissue glucose and lipid metabolism. Advances inmulti-omics approaches are expected to provide deeper insights into the pathogenesis of vascular aging, although significant challenges remain. This review aims to advance understanding of the pathogenesis of vascular aging and to guide novel therapeutic strategies, particularly for older adults at high risk.

Research into the molecular mechanisms linking glucose-lipid metabolism and vascular aging has demonstrated that vascular aging involves multiple metabolic pathways and cellular functions. Future directions hold promise for the development of precision medicines targeting specific metabolic pathways, along with multi-target combination therapies. Advancements in multi-omics technologies are providing a more comprehensive understanding of the specific mechanisms underlying vascular aging and informing corresponding intervention strategies. Integrated multi-omics technologies are also expected to facilitate the early identification and diagnosis of vascular aging, thereby supporting the timely implementation of preventive measures. Within the framework of individualized precision medicine, tools such as genomics and metabolomics can elucidate patient-specific pathogenic mechanisms. These insights may guide personalized treatment strategies that encompass diet modifications, exercise, medication, and patient education to achieve optimal therapeutic outcomes.
